# Umbilical cord blood exosomes from very preterm infants with bronchopulmonary dysplasia aggravate lung injury in mice

**DOI:** 10.1038/s41598-023-35620-8

**Published:** 2023-05-27

**Authors:** Xin-qi Zhong, Tao-fang Hao, Qi-jiong Zhu, Jing Zheng, Mao-fei Zheng, Xiu-hong Li, Li-hua Luo, Chang-shun Xia, Yu-wei Fan, Jian Gu, Tao Liu, Dun-jin Chen

**Affiliations:** 1 Department of Neonatology, The Third Affiliated Hospital of Guangzhou Medical University, 63 Duobao Road, Liwan District, Guangzhou, 510150 China; 2 Key Laboratory for Major Obstetric Disease of Guangdong Province, Guangzhou, China; 3 Department of Pharmacology, Zhongshan School of Medicine, Sun Yat-Sen University, Guangzhou, China; 4 Department of Public Health and Preventive Medicine, School of Medicine, Jinan University, Guangzhou, 510632 China; 5 China Greater Bay Area Research Center of Environmental Health, School of Medicine, Jinan University, Guangzhou, China; 6 Department of Obstetrics and Gynecology, University of WI–Madison, Madison, WI USA; 7 Department of Maternal and Child Health, School of Public Health, Sun Yat-Sen University, Guangzhou, China; 8 Department of Obstetrics and Gynecology, The Third Affiliated Hospital of Guangzhou Medical University, Guangzhou, 510150 Guangdong China

**Keywords:** Molecular biology, Respiratory tract diseases

## Abstract

Bronchopulmonary dysplasia (BPD) is characterized by abnormal development of the blood vessels and alveoli in lungs, which largely occurs in premature infants. Exosomes (EXO) from very preterm infants (VPI) with BPD (BPD-EXO) impair angiogenic activities of human umbilical vein endothelial cells (HUVECs) via EXO-miRNAs cargo. This study aimed to determine whether and how BPD-EXO affect the development of BPD in a mouse model. We showed that treating BPD mice with BPD-EXO chronically and irreversibly aggravated lung injury. BPD-EXO up-regulated 139 and down-regulated 735 genes in the mouse lung tissue. These differentially expressed genes were enriched to the MAPK pathway (e.g., *Fgf9* and *Cacna2d3*), which is critical to angiogenesis and vascular remodeling. BPD-EXO suppressed expression of *Fgf9* and *Cacna2d3* in HUVECs and inhibited migration, tube formation, and increased cell apoptosis in HUVECs. These data demonstrate that BPD-EXO aggravate lung injury in BPD mice and impair lung angiogenesis, plausibly leading to adverse outcomes of VPI with BPD. These data also suggest that BPD-EXO could serve as promising targets for predicting and treating BPD.

## Introduction

Nearly 15 million babies are born preterm before 37 weeks of gestation annually worldwide, which is a major global healthcare problem^1^. Among preterm birth, 10% with gestational age of less than 32 weeks are defined as very preterm infants (VPI) who are at greater risk of short- and long-term health problems.

To date, the improved perinatal care has greatly increased the survival rate of VPI^2^. However, approximately 40% of VPI still develop bronchopulmonary dysplasia (BPD), placing a huge financial burden on families and healthcare as children require medical intervention^3,4^. BPD can be resulted from either prenatal injury or repeated postnatal injuries in the developing lung. As a result, lung development is significantly defective, leading to persistent airway and pulmonary vascular dysfunction^5^. Despite the application of various means of respiratory care, such as mechanical ventilation and corticosteroids, BPD remains the most common and serious complication in VPI^5^. Thus, understanding the mechanisms underlying BPD and lung repairing will help us further improve the outcome of premature infants.

The main pathological phenotype of BPD includes arrested alveolarization and microvascular growth^5,6^. Increasing evidence supports that supplemental oxygen alone actually can cause BPD in animal models^7^. The disordered expression of angiogenic factors and their receptors is also associated with BPD-impaired function of pulmonary microvascular. Vascular endothelial growth factor-A (VEGFA) is one of several angiogenic factors that play a central role in the development and function of arteries and veins^8,9^. Furthermore, the disruption of VEGFA signaling contributes to the pathogenesis of BPD by impairing lung vascular growth. Recently, it has been reported that mesenchymal stromal cells (MSC)-derived exosomes can regulate VEGFA and angiogenesis as a potential drug for BPD treatment, but lung fibroblast-derived exosomes did not show any benefit in hyperoxia-induced mice^10,11^. This suggests that exosomes from different sources may have different effects on BPD.

Exosomes (EXO) are small extracellular vesicles with a diameter ranging from 30 to 120 nm^12,13^. Exosomes from MSC can strengthen endothelial cell tube formation in vitro, and vascularization and alveolarization *in vivo*^14^. Exosomes from endothelial progenitor cells can stimulate angiogenesis of pulmonary microvascular endothelial cells^15^. Exosomes from lung spheroid cells can also promote lung repair in pulmonary fibrosis^16^. An exosome-associated gene, fibroblast growth factor 9 (FGF9), could regulate both capillary plexus formation and VEGFA expression, which is required for early lung distal vascular development^17–19^. We have recently reported that umbilical cord blood-derived EXO from VPI with BPD inhibit angiogenesis in vitro by miRNAs (e.g., miR-185-5p)^20^. However, it remains elusive whether BPD-EXO suppress pulmonary angiogenesis or development in vivo.

Here, we verified the effect of BPD-EXO in a mouse model of hyperoxia-induced BPD and validated our hypothesis that BPD-EXO could aggravate hyperoxia-induced lung injury. At the same time, we used RNA-seq to find that the differentially expressed genes in the lung tissue of BPD mice treated with BPD-EXO were related to the MAPK signalling pathway, and verified that it could affect angiogenesis in vitro These findings are more helpful to our comprehensive understanding of BPD-EXO and better discovery of targets for the treatment of BPD.

## Methods

All experimental procedures were designed and performed following standard guidelines of the Third Affiliated Hospital of Guangzhou Medical University and the ARRIVE guidelines.

### Exosome isolation and identification

The recruitment criteria for patients with very preterm delivery (28–31^+6^ weeks) were previously described^20^. All procedures were conducted in accordance with the Declaration of Helsinki. The umbilical cord blood collection protocol was approved by the Institutional Review Board of the Third Affiliated Hospital of Guangzhou Medical University (Protocol # 2021–014). All subjects gave written, informed consent before participating. Clinical diagnosis of healthy pregnancy was made by the obstetricians at the birth center (, the Third Affiliated Hospital of Guangzhou Medical University, where the umbilical cords were collected. Exosomes were obtained by ultracentrifugation of serum from extremely premature infants (2 ml/infant) as we described previously^20^.

Exosome suspension (5 μl/infant mixed with 5 μl H_2_O) was dropped to the copper mesh for 1 min and stained with 10 μl phosphotungstic acid solution for 1 min. Morphology of exosomes was observed by a transmission electron microscope (JEOL-JEM1400, JEOL Ltd., United States) at an acceleration voltage of 80 kV. Exosome suspension (5 μl/infant mixed with 25 μl H_2_O) was used to determine the size distribution of exosomes by nanoparticle tracking analysis (NTA) using a Particle Metrix ZetaView instrument (Particle Metrix GmbH, Germany) with laser and video camera module (Particle Metrix GmbH, Germany). Exosome surface marker proteins TSG101 and Alix were determined by western blot.

### Western blot analysis

Exosomes were lysed with RIPA buffer (Thermo Scientific) following the manufacturer's instructions. Quantity was determined with the BCA kit (Thermo Scientific). Equal amounts of proteins were subjected to Western blotting analysis as described previously^18^. The proteins on membranes were probed using antibody against Alix (CST, #2171, 1:1000) or TSG101 (Abcam, #ab125011, 1:5000). The protein signal was detected using Chemiluminescent ECL reagent (Forevergen, China).

### Construction of a mouse model of bronchopulmonary dysplasia

Pregnant (8-week-old) SPF FVB/NJ mice were purchased from Beijing Huafukang Biotechnology Co., Beijing, China. Thirty newborn mice were randomly divided into five groups (n = 6/group). One group was kept in an ambient condition (~ 21% O_2_) as the normoxia group. Other four groups of mice were transferred to a 75% O_2_ (hyperoxia) environment from PND 2 to 14. After 2 days exposure to hyperoxia, mice were subsequently divided into 4 subgroups, one of which was used as a control group and was injected with only PBS, and the other three groups injected intraperitoneally with 16.67 μl BPD-EXO at concentrations of 1 × 10^4^, 1 × 10^5^, and 1 × 10^6^ particles/μl per day from PND 4 to 6 for each mouse, respectively, and the total injection volume was 50 μl for three days of injection. Then, all 30 mice were sacrificed on PND14 after parturition and the lung tissues were dissected for pathological examination.

To determine the chronical effects of hyperoxia and BPD-EXO on lung development, eighteen PND1 mice were randomly divided into three groups (n = 6/group). Group 1 was kept under ~ 21% O_2_ from PND 1 to 42 as the normoxia group. Groups 2 and 3 was transferred to a 75% O_2_ (hyperoxia) environment from PND 2 to 14 and then returned to normoxia up to PND42. Groups 3 mice were daily injected intraperitoneally with 16.67 μl BPD-EXO at 1 × 10^4^ particles/μl per mouse from PND 4 to 6. Mice in all groups did not die until the 42nd day for sacrifice.

All procedures were approved by the Institutional Animal Care and Use Committee of the Third Affiliated Hospital of Guangzhou Medical University (Protocol # 2019–188). Animals were maintained and treated in the Animal Centre of Guangzhou Medical University according to the rules of Committee on Animal Research and Ethics.

### Hematoxylin & eosin staining (H&E) and Masson's trichrome staining

The lung tissues were fixed with 10% formalin solution in 4℃ for 24 h and embedded in paraffin. The tissue blocks were sliced (5 μm thickness), dewaxed stained with H&E. The slide images were recorded using a Nikon Eclipse CI microscopy (Nikon, Japan). Additional serial sections were stained with Masson's trichrome according to routine procedures^21^ to visualize and quantify tissue collagen.

### Immunohistochemistry (IHC) for VEGFA and CD31

IHC for VEGFA and CD31 was performed on lung tissue sections as described^20^. The deparaffined sections were probed with mouse anti-VEGFA (Boster, BA0407, 1:200) or rabbit anti-CD31 (Abcam, ab28364, 1:50), followed by the second antibody conjugated with Horseradish peroxidase (HRP) (Boster, SV0001). The staining color was developed using a DAB Substrate Kit (Abcam, ab64238), and counterstained with hematoxylin. Positive results were shown as brownish yellow. PBS missing the primary antibody was used as the negative staining control. The images were observed and recorded by a Nikon Eclipse CI microscopy (Nikon, Japan). Staining density was determined and quantified with Image J software using 20 × or 40 × images^22^.

### Histopathology scores

Semi-quantitative histopathology scoring criteria were described by Shen et al.^23,24^. Lung injury was evaluated based on lung edema, infiltration of inflammatory cells, alveolar hemorrhage, hyaline membrane, and atelectasis. The scores were determined based on the area of injured: no lesion, score 0; injured area ≤ 25%, score 1; injured area 26–50%, score 2; injured area 51–75%, score 3; injured area > 75%, score 4. The scores were averaged for each animal from 10 randomly selected fields.

### RNA-seq and bioinformatics analysis

Total RNA was extracted from tissues using TRIzol reagent (Invitrogen, Carlsbad, CA) according to the manufacturer's instructions. The RNA concentration and purity were determined by a Nanodrop2000 spectrophotometer (Thermo Scientific, US). The samples with OD^260:280^ around 2.0 were further measured the RNA integrity number (RIN) using Agilent 2100 bioanalyzer (Agilent, US). The RNA samples with RIN higher than 7 were enriched mRNA with magnetic beads with Oligo(dT) and performed the library construction using NEBNext® Ultra™ II RNA Library Prep Kit for Illumina (E7770, NEB, US). The libraries were qualified by Agilent 2100 Bioanalyzer and sequenced with 150 bp paired end on Illumina HiSeq2000 (Illumina, US). The resulting data were submitted to the Short Read Archive (NCBI) database (AccessionID: PRJNA917079).

The sequencing reads were filtered at low quality and trimmed the adaptors using Trim Galore software (v0.6.7)^25^. The resulting high-quality reads were aligned to the Mus musculus reference genome GRCm39 by Hisat2 (v2.2.1)^26^, and generated the gene reads count matrix using featureCounts^27^. Differential expressions between conditions were assessed based on the negative binomial distribution using DESeq2^28^. The differentially expressed genes (DEGs) with FDR ≤ 0.001 and Log2(Fold Change) ≥ 1 were used to perform GO and KEGG pathway enrichment analysis using clusterProfiler package (v4.0.5) in R (v4.0.2)^29^.

### Quantitative reverse transcription PCR (RT-qPCR)

Total RNA was digested to remove DNA contaminations by Turbo DNase (Invitrogen, US), followed by reverse transcribed using the Reverse Transcription System (Promega, US) according to the manufacturer’s instructions. Quantitative PCR was performed using SuperReal SYBR Green PreMix (Tiangen Biotechnology, China) in the ABI PRISM 7500 System (Thermo Scientific, US). Relative gene expression was evaluated using 2^-△△Ct^ methods^30^. The housekeeping gene GAPDH was used for normalization. The primers used in this study were attached in Table S2.

### Cell culture

Human umbilical vein endothelial cells (HUVECs) were purchased (Cellcook, Guangzhou, China). A high-glucose medium of Dulbecco's Modified Eagle Medium (DMEM, Gibco, US) containing 10% fetal bovine serum (FBS, Gibco, US) was used for maintaining HUVECs. Cells were then cultured at 37 ℃ with 5% CO_2_. Before treated with exosomes (100 μg/ml), cells were passaged and serum-starved for 16 h, followed by carrying out cell migration, tube formation, and cell apoptosis assays.

### Cell migration assay

HUVECs migration was tested by transwell chambers (8-μm pore size, Corning, US) based on the manufacturer’s instructions. HUVECs suspended in a serum-free medium (1 × 10^5^ cells) was seeded to each wells of the upper chamber. The complete medium (600 μl/well) were added into the lower chambers. After 24 h culture, the migrated cells were fixed with 4% paraformaldehyde for 15 min and stained with 1% crystal violet (Beyotime Biotechnology, Beijing, China) for 10 min. Cells in five random separate microscope fields were captured using a Nikon Eclipse Ti microscope (Tokyo, Japan) and counted (200 × magnification) using Image J software^22^.

### Tube formation assay

As described by the manufacturer’s instructions, 48 well plates were coated with Matrigel (BD Biosciences, US). HUVECs were digested and diluted to the concentration of 8 × 10^5^ cells/ml, and then 200 μl cells supernatant were inoculated to Matrigel-coated 48-well plates and treated with exosomes. Cells were then incubated at 37 ℃ with 5% CO_2_ Up to 8 h Three random fields were captured under a microscope (Olympus BX51, Japan) every two hours after treatments, and the tube formation was analyzed with Image J software^22^.

### Flow cytometry analysis of cell apoptosis

The apoptosis was measured using the Dead Cell Apoptosis Kit with Annexin V Alexa Fluor 488 & Propidium Iodide (PI) (Invitrogen, US). Briefly, after HUVECs (1 × 10^6^ cells/well) were treated with exosomes (200 μg) for 48 h, cells were washed with PBS and suspended in 1 × Annexin-binding buffer. Then, cells were stained with 5 μl Annexin V-Alexa Fluor 488 and 7 μl PI for 15 min at room temperature. The apoptosis was determined by flow cytometer (FACSAria III, BD, USA) and Flowjo software.

### Statistical analysis

All quantitative data were presented as Mean ± SD. The differences between two groups were analyzed with the unpaired Student’s t-test with *p-value* < 0.05 (*) or < 0.01 (**). The statistically significant differences between the means of three or more groups were compared with One-way ANOVA followed by Bonferroni’s post hoc test, and the different letters were used to show statistical significance (*p-value* < 0.05 (*) or < 0.01 (**)). All statistical analyses were carried out using SPSS 22 software.

## Results

### Isolation and identification of exosomes from VPI with BPD

**Figure 1:**
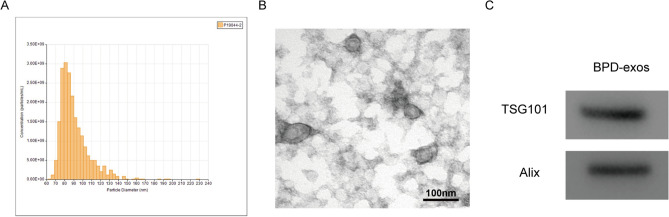
Characterization of the isolated exosomes by NAT, TEM, and WB. (**A**) NAT. Sizes distribution of the isolated exosomes. (**B**) Morphology of negative-stained exosomes under TEM. (**C**) WB. Expression of exosomes surface markers TSG101 and Alix. n = 3.

Nanoparticles were isolated from the patients of very preterm infants with BPD as described^20^. The isolated nanoparticles were analyzed by transmission electron microscopy (TEM), nanoparticle tracking analysis (NTA), and western blot (WB). Diameters of nanoparticles ranged from 30 to 150 nm (Fig. 1A) with an oval or round vesicle-like structure and a negative-stain lipid bilayer membrane (Fig. 1B). The exosome-surface markers, TSG101 and Alix were both positively detected in the vesicles (Fig. 1C). All these results confirmed that the nanoparticles were exosomes.

### BPD-EXO aggravate lung injury in BPD mice

**Figure 2:**
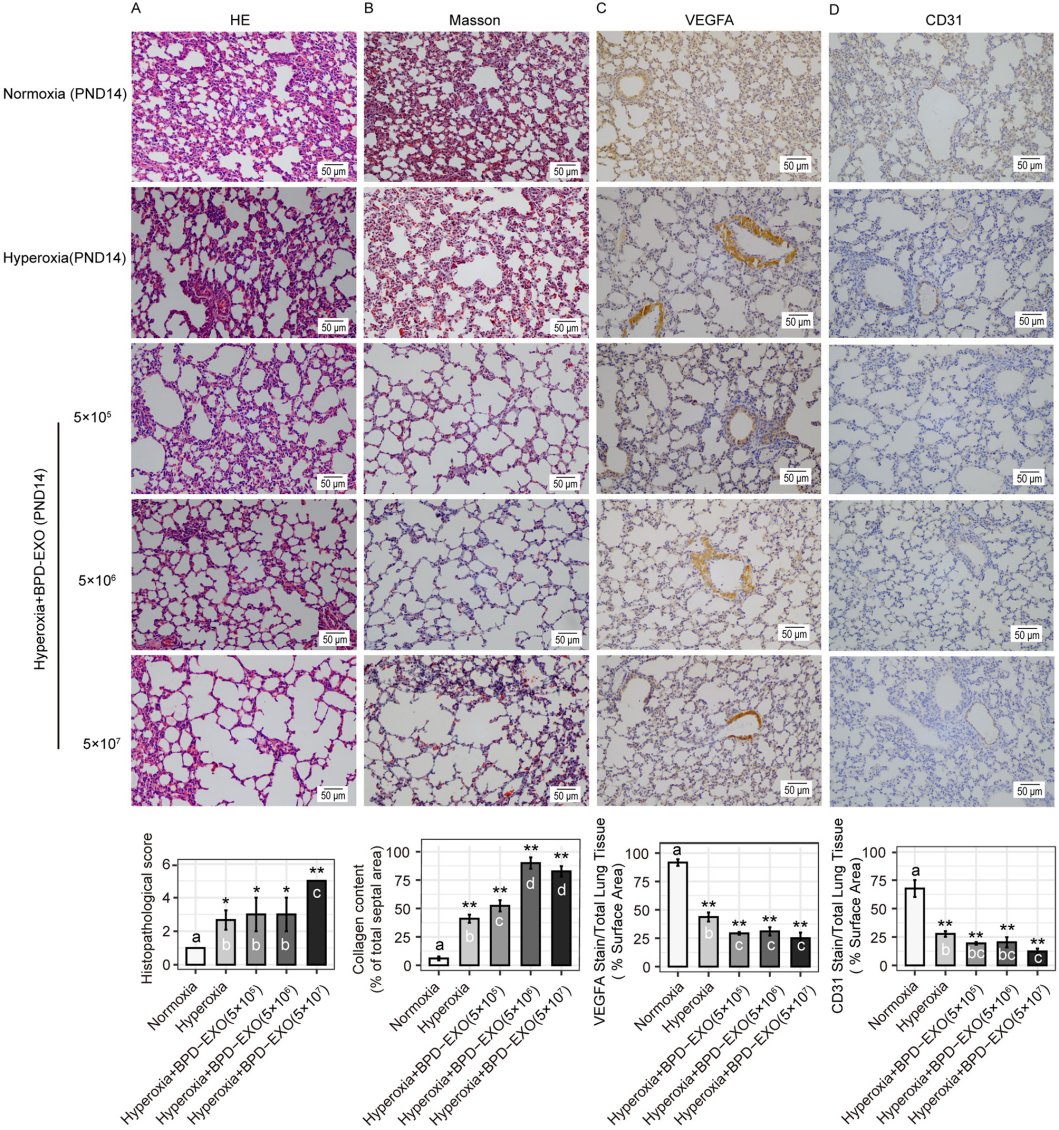
BPD-EXO aggravate lung injuries and angiogenesis impairment in BPD mice. (**A**) H&E staining. Lung tissue sections were stained with H&E and the histopathology scores were determined. (**B**) Mass staining. Lung tissue sections were stained with Masson and the collagen content were assessed. (**C**) Immunohistochemical staining of VEGFA (**C**) and CD31 (**D**). Lung tissue sections were immunostained for VEGFA and CD31. Representative images are shown. BPD, bronchopulmonary dysplasia; BPD-EXO, umbilical cord blood-derived exosomes from very preterm infants with BPD. PND, postnatal day; VEGFA, vascular endothelial growth factor A; CD31, cluster of differentiation 31. Scale bar = 50 μm. All data are presented as the mean ± SD. One-way ANOVA followed by Bonferroni’s post hoc test, and the different letters indicate statistical significance. n = 6. *, *P* < 0.05; **,. *P* < 0.01.

The H&E staining showed that compared with normoxia, hyperoxia induced BPD-like histocytology changes in the lung tissues (Fig. 2). Specifically, hyperoxia enlarged distal airspaces with fewer separation, reduced alveolarization, and increased alveolar interstitial cells in lung tissues (Fig. 2A). In addition, hyperoxia increased pulmonary fibrosis, decreased density of pulmonary microvessels, and dilated pulmonary capillaries based on the Masson staining. Compared with normoxia, hyperoxia increased (*P* < 0.05) collagen content (Fig. 2B). To detect the expression of VEGFA and CD31, the latter of which is an endothelial marker, we performed immunohistochemistry (IHC) in the lung tissue section. The IHC analysis revealed that staining areas of VEGFA and CD31 in hyperoxia group were decreased (*P* < 0.05) compared to normoxia group (Fig. 2C,D). These results demonstrate the successful establishment of the BPD animal model.

On PND14, compared with hyperoxia, 5 × 10^7^ BPD-EXO particles further increased (*P* < 0.05) pulmonary alveolar fusion and fibrosis, causing a significant increase in the histopathology scores (Fig. 2A,B). Compared to hyperoxia-treated group, BPD-EXO at 5 × 10^5^, 5 × 10^6^, and 5 × 10^7^ particles treated group also increased (*P* < 0.05) collagen content (Fig. 2A,B). Compared with hyperoxia, BPD-EXO at 5 × 10^5^, 5 × 10^6^, and 5 × 10^7^ particles decreased (*P* < 0.05) the VEGFA and CD31 staining distribution (Fig. 2C,D). Taken together, these data indicate that BPD-EXO exaggerates lung tissue injury and impairs lung angiogenesis in BPD mice.

### BPD-EXO chronically aggravate lung injury in BPD mice

**Figure 3:**
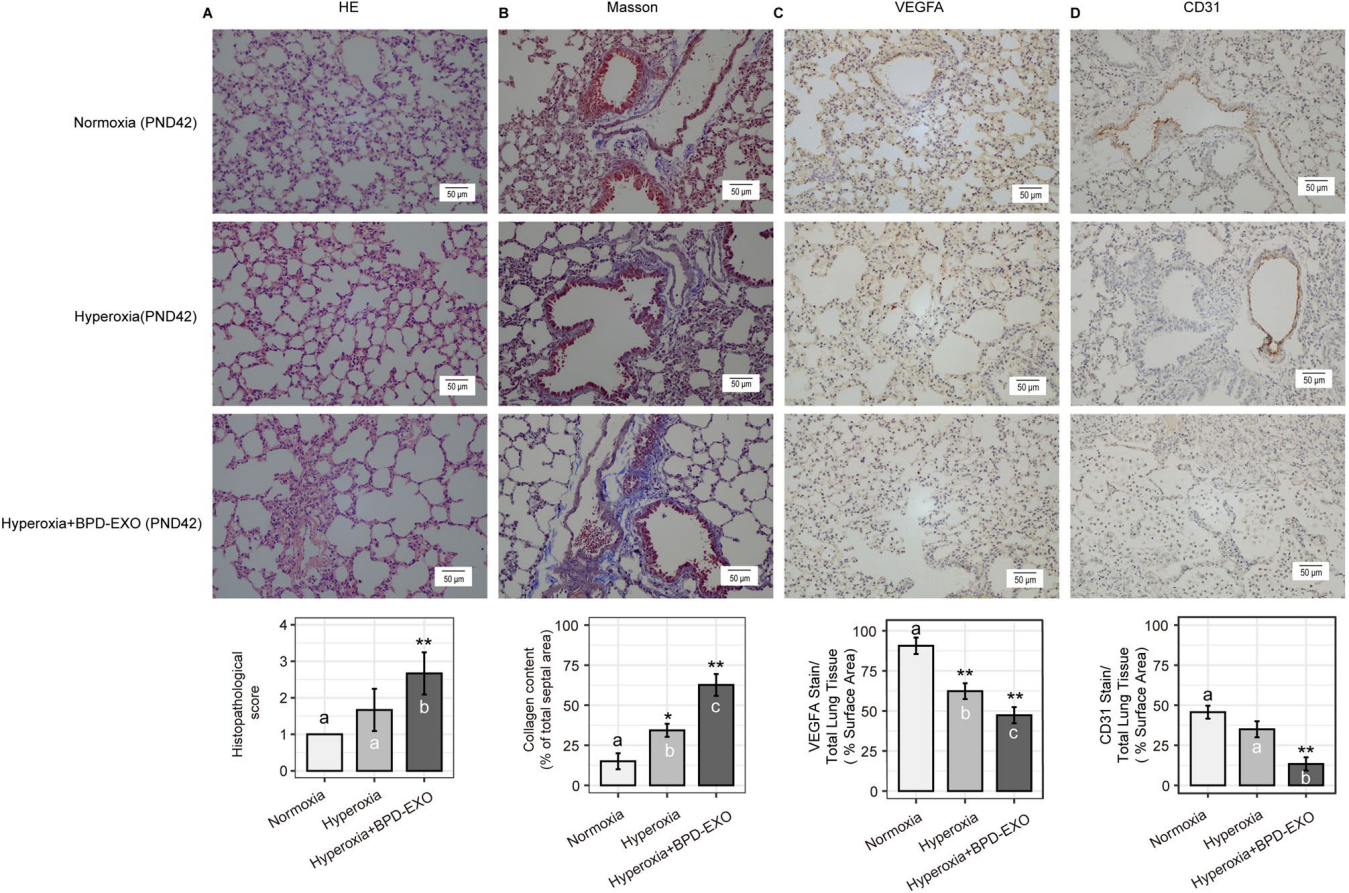
Re-normoxia does not to reverse BPD-EXO- aggravated pulmonary damage. One-day-old mice were exposed to hyperoxia for 14 days then return to normoxia for 28 days. The lung tissue sections were stained with H&E (**A**) and Masson (**B**) to quantify histopathology scores (**A**) and collagen content (**B**), respectively. Additional tissue sections were immunostained for VEGFA (**C**) and CD31 (**D**) to quantify the expression. Representative images are shown. PND, postnatal day; VEGFA, vascular endothelial growth factor A; CD31, cluster of differentiation 31. Scale bar, 50 μm. All data are presented as the mean ± SD. n = 6. One-way ANOVA followed by Bonferroni’s post hoc test, and the different letters were used to show statistical significance (*P < 0.05; **P < 0.01).

After 28 days of normoxia, the hyperoxia-increased septum, enlarged distal cavity, reduced complexity, and increased pulmonary fibrosis persisted (Fig. 3A,B) (*P* < 0.05). Compared to nomoxia, hyperoxia decreased staining areas of VEGFA and CD31 (*P* < 0.05) (Fig. 3C,D). These results suggest that hyperoxia-induced BPD in neonatal mice can influence the lung development late in life. To determine the chronic effects of BPD-EXO on PBD mice, exposure of hyperoxia-treated mice from PND 4 to 6 to BPD-EXO (5 × 10^5^ particles) increased pulmonary damage and fibrosis (Fig. 3A,B). Compared with hyperoxia, BPD-EXO at 5 × 10^5^ particles decreased (*P* < 0.05) the VEGFA and CD31 staining distribution (Fig. 3C,D). From the above results, we confirmed that BPD-EXO has an irreversible aggravating effect on lung injuries of hyperoxia-induced rats and the adverse effects can last up to the sexually mature stage.

### BPD-EXO alter gene expression in lung tissues of BPD mice

**Figure 4:**
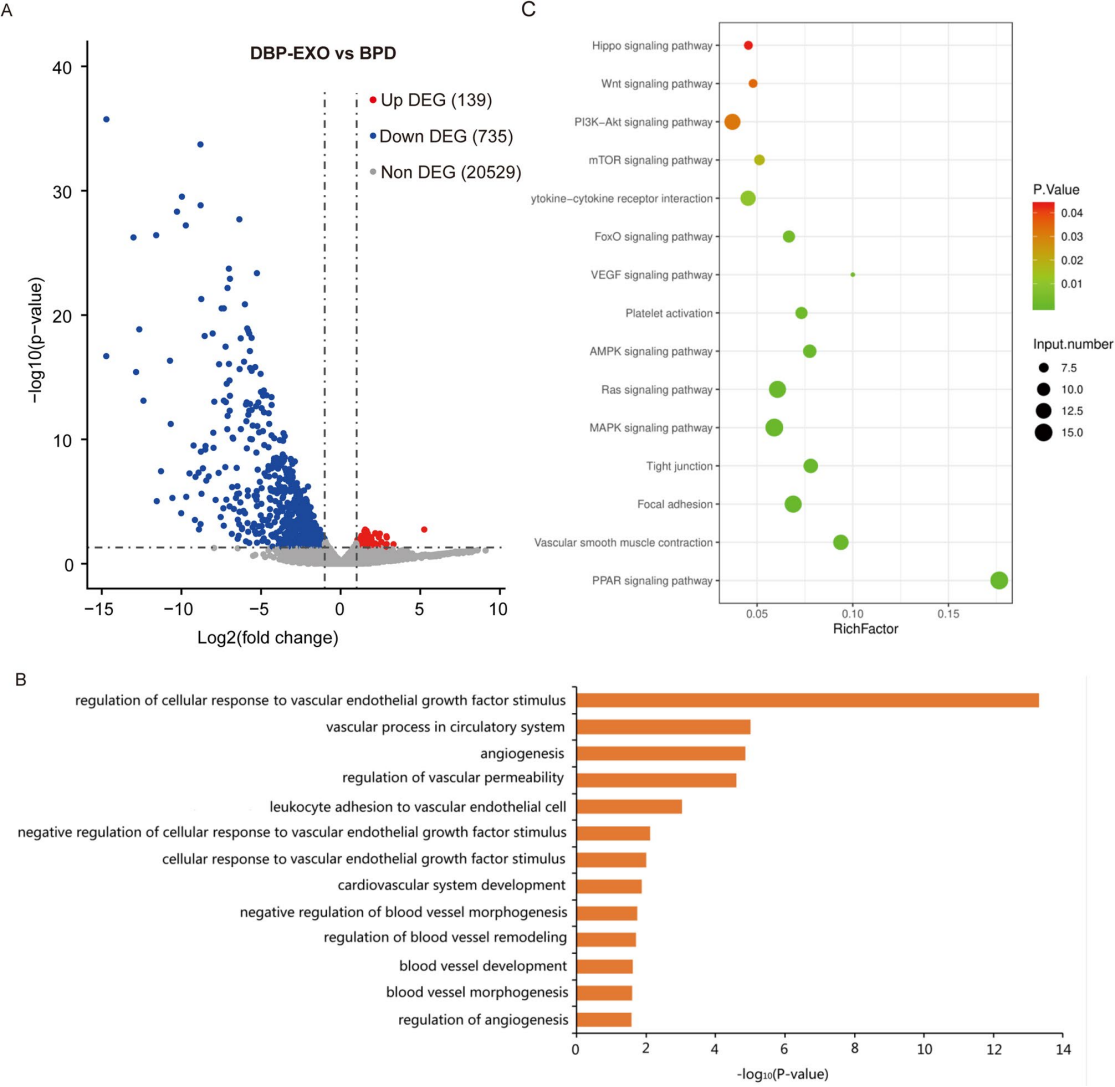
Differential gene expression profile, gene ontology, and pathway enrichment analysis for lung tissues from BPD mice treated with BPD-EXO or vehicle. (**A**) The volcano plot of the differential gene expression profile was determined by RNA-seq. (**B**) The gene ontology (GO) enrichment analysis of the biological process. (**C**) KEGG pathway enrichment analysis identified the top 15 pathways affected by BPD-EXO.

More than 196 million reads were trimmed adaptor and passed the quality filter. Clean reads were aligned to the mouse genome (GRCm39/mm39) using HISAT2^26^, with a mean alignment rate of more than 96.0% (Table S1). A total of 874 BPD-EXO-induced DEGs were identified, of which 139 were up-regulated and 735 were down-regulated in the BPD-EXO-treated lung tissues (Fig. 4A).

GO analysis revealed that these DEGs were significantly enriched in biological processes closely related to vascular formation, remodeling, and development, such as regulation of cellular response to vascular endothelial growth factor stimulus, vascular process in circulatory system, and angiogenesis (Fig. 4B). KEGG pathway enrichment analysis found that the DEGs were mainly enriched in signaling pathways involved in the function of angiogenesis and vascular remodeling, including peroxisome proliferator-activated receptors (PPAR) signaling pathway, vascular smooth muscle contraction, focal adhesion, tight junction, mitogen-activated protein kinase (MAPK) signaling pathway, Ras signaling pathway, AMP-activated protein kinase (AMPK) signaling pathway, etc. (Fig. 4C).

**Figure 5:**
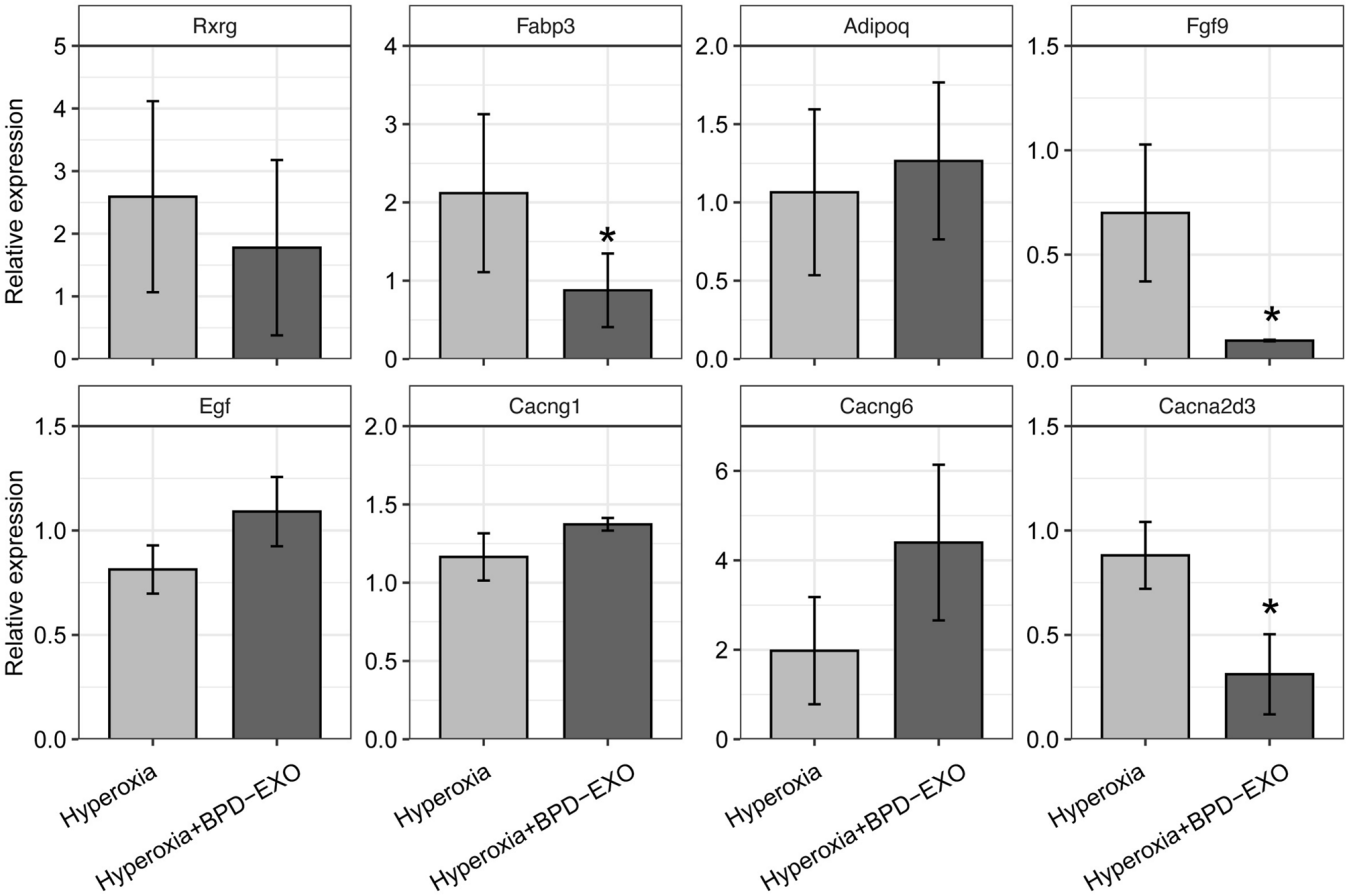
Validation of DEGs expression by RT-qPCR. Differential expression of genes in PPAR signaling pathway (*Rxrg*, *Fabp3*, *Adipoq*) and MAPK pathway (*Cacng1*, *Cacng6*, *Egf*, *Cacna2d3*, *Fgf9*) between pulmonary samples collected from BPD mice and BPD-EXO treated BPD mice. n = 4. *P < 0.05.

Among these DEGs identified, the top 15 enriched signaling pathways, PPAR and MAPK pathways are related to angiogenesis and vascular remodeling^31,32^. The PPAR signaling pathway was enriched with the genes of *Rxrg*, *Fabp3*, and *Adipoq* genes, and the MAPK pathway was enriched with genes of C*acng1*, *Cacng6, Egf, Cacna2d3,* and *Fgf9.* We verified the expression of these genes in lung samples of BPD mice and BPD-Exon-treated BPD mice by RT-qPCR. In line with the transcription results, RT-qPCR results showed that *Fabp3*, *Fgf9*, and *Cacna2d3* were significantly down-regulated in the BPD-EXO treated BPD mice (Fig. 5).

### BPD-EXO inhibit endothelial cell angiogenesis, and promote endothelial cell apoptosis by regulating the MAPK signaling pathway in HUVECs

**Figure 6:**
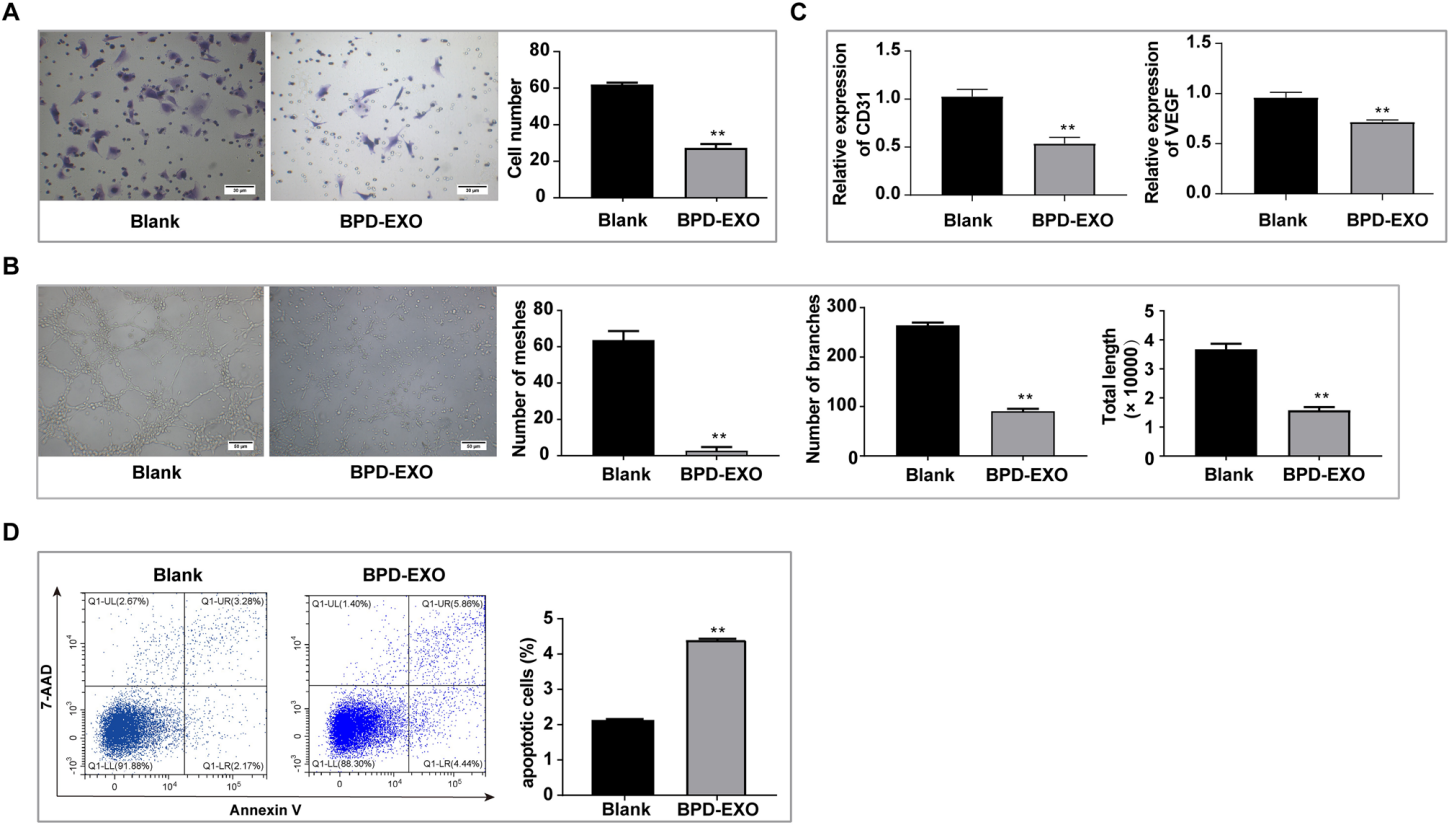
Effects of BPD-EXO on endothelial angiogenesis and apoptosis. HUVECs were treated with BPD-exosomes (100 μg/ml) or vehicle control for 48 h under 21% O_2_ conditions. (**A**) Migrated cells were quantified by transwell assay. (**B**) Tube formation was quantified by measuring tubes per well using ImageJ software. (**C**) Expression of CD31 and VEGFA were quantified by RT-qPCR. (**D**) Cell apoptosis was determined using flow cytometry. Scale bar = 30 μm. n = 3. **P < 0.01.

Compared with the negative control, BPD-EXO significantly inhibited (*P* < 0.05) endothelial cell migration and tube formation (Fig. 6A,B). Compared with vehicle control, BPD-EXO reduced (*P* < 0.01) mRNA expression of CD31 and VEGFA (Fig. 6C). BPD-EXO also increased (*P* < 0.05) the number of apoptotic cells in BPD-EXO group compared with the vehicle control group) (Fig. 6D).

**Figure 7:**
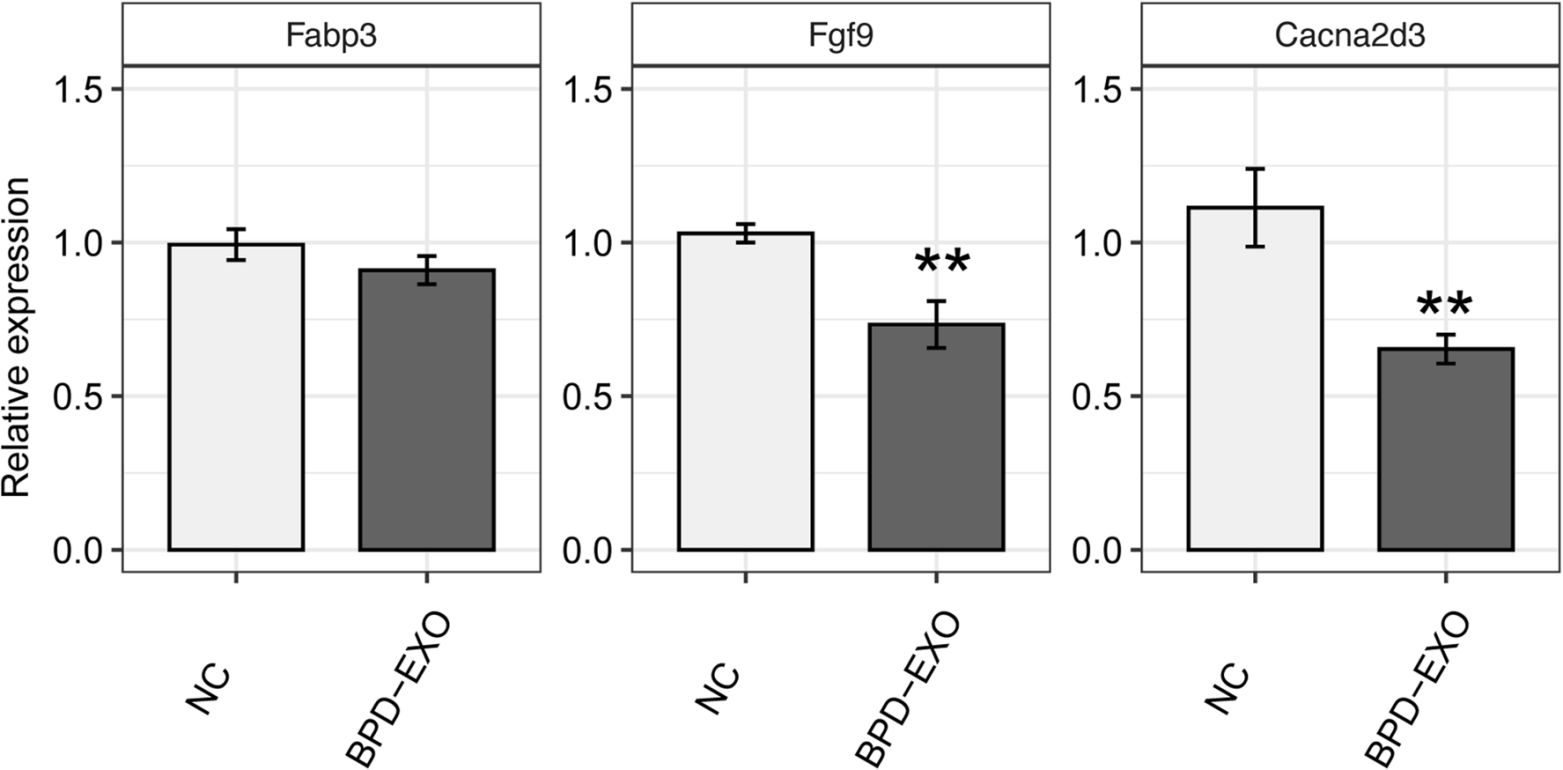
BPD-EXO suppress gene expression involved in the MAPK pathway in HUVECs. Cells were treated with BPD-EXO or vehicle control for 48 h. Expressions of *Fabp3* (**A**), *Fgf9* (**B**), and *Cacna2d3* (**C**) were determined by RT-qPCR. n = 3. **P < 0.01.

We further verify the effects of BPD-EXO on mRNA expression of *Fabp3*, *Fgf9*, and *Cacna2d3* in HUVECs. The RT-qPCR results showed that BPD-EXO significantly decreased expression of *Fgf9* and *Cacna2d3*, but not *Fabp3* (Fig. 7), which was in line with the results in pulmonary tissues (Fig. 5). As *Fgf9* and *Cacna2d3* both belong to the MAPK signaling pathway, it suggests that BPD-EXO may impair endothelial functions by dysregulating the MAPK signaling pathway.

## Discussion

In the present study, we have demonstrated: 1) BPD-EXO aggravate lung injury and impair lung angiogenesis in a hyperoxia mouse model; 2) we have further shown that the adverse effect of BPD-EXO on lung tissue and angiogenesis in neonate can persist in adult mice; 3) many BPD-EXO-induced DEGs and pathways may contribute to these adverse effects on lung tissues and vascular growth; and 4) BPD-EXO inhibit angiogenesis in vitro in association with decreased *fgf9* and *Cacna2d3* in HUVECs. These data suggest that BPD-EXOs may contribute to the development of BPD and BPD-related lung disease, and BPD-EXO and their downstream genes and pathways could serve as the predictive biomarkers and therapeutic targets for VPI with BPD.

Our previous study showed that BPD-EXO impairs angiogenesis in HUVECs^20^, but whether BPD-EXO has such an effect in vivo needs further verification. Gareth R et al. found that exosomes derived from human MSC medium supernatant can alleviate hyperoxia-induced lung tissue damage in neonatal mice^33^. Liping Teng et al. also reported that exosomes secreted by umbilical cord blood-derived MSCs can promote the proliferation HUVECs and angiogenesis^34^. The exosomes secreted by these umbilical cord blood MSCs can regulate related diseases through miRNA, regulate proliferation and migration-related TGF-β signaling pathways^35^, and also regulate inflammation-related GSK3β/Wnt/β^36^ and IL-6/STAT3 pathway^37^. Umbilical cord blood exosomes are primarily protective in lung disease^38^. Another study of ours also showed that exosomes derived from healthy term umbilical cord blood can alleviate hyperoxia-induced lung injury^39^. But in the present study, we found that treatment with cord blood exosomes from BPD aggravated hyperoxia-induced lung injury. This discrepancy may be due to the heterogeneity of exosomes from different sources or severities of the same disease may exhibit different actions^12,40^. For example, it is possible that only umbilical cord blood EXO from VPI but not other preterm infants with BPDs can cause the aggravating of hyperoxia-induced lung injury.

In the current study, we used two mouse models of hyperoxia injury to evaluate the effect of BPD-EXO on hyperoxia-induced lung injury, short-term hyperoxia-induced and long-term hyperoxia-induced. Mice's lung development is largely completed by 2–3 weeks after birth^41^. Five days after birth, the lungs of the mice were still saccular, and complete alveoli were not formed until the 30th-day^42,43^. Therefore, it is best to choose a short-term model to study the damage of hyperoxia to alveoli, that is, induce hyperoxia for 14 days. Hyperoxia-induced injury is associated with long-term pulmonary complications such as secondary pulmonary hypertension (PH)^33^. We chose to continue to observe the 42nd day after hyperoxia induction to evaluate mice's lung injury after hyperoxia induction in adulthood. Our results indicated that hyperoxia-induced damage was more severe in BPD-EXO-treated mice, regardless of the early model or the long-term model. However, further experiments are needed to verify whether BPD-EXO treatment is more serious for hyperoxia-induced early lung development damage or longer-term lung damage.

The current observation that BPD-EXO down-regulated *Fgf9* and *Cacna2d3* gene expression both in vitro and in vivo is not surprising since both of these two genes are enriched in the MAPK signaling pathway and are closely related to angiogenesis^44^. Particularly, *Fgf9* could regulate both capillary plexus formation and VEGFA expression in endothelial cells, which is required for early lung distal vascular development^17–19^. *Cacna2d3* regulates cell apoptosis and cell replication through the MAPK pathway and PI3K/AKT pathway, both of which are associated with angiogenesis^45^. The human and mouse FGF9 3’ UTR are highly conserved and are similarly regulated by miR-182 and miR-328. Interestingly, both miR-183 and miR-328 were up-regulated EXO-miRNA in the BPD-EXO^20,46,47^. *Cacna2d3* are target genes of miR-27a which was the up-regulated EXO-miRNA in the BPD samples^20,48^. These findings suggest that EXO could be used as treatment options for BPD, since their involvements in the pathogenesis of BPD. Preterm infant-derived exosomes are a possible cause of BPD progression and regulating molecule cargo of EXO might rescue BPD-impaired fetal angiogenesis and promote lung development. Future investigations are needed to be performed on the role of specific molecules in exosomes on BPD progression and to provide more clinical and research evidence for innovative diagnosis tools and therapy methods for VPI with BPD.

## Supplementary Information

Supplementary Information 1.

Supplementary Information 2.

Supplementary Information 3.

## Abbreviations

AMPK: AMP-activated protein kinase
BPD: Bronchopulmonary dysplasia
BPD-EXO: Exosomes from very preterm infants with Bronchopulmonary dysplasia
DEGs: Differentially expressed genes
EXO: Exosomes
FGF9: Fibroblast growth factor 9
H&E: Hematoxylin & eosin staining
HUVECs: Human umbilical vein endothelial cells
IHC: Immunohistochemistry
MAPK: Mitogen-activated protein kinase
MSC: Mesenchymal stromal cells
NTA: Nanoparticle tracking analysis
PI: Propidium Iodide
PPAR: Peroxisome proliferator-activated receptors
RT-qPCR: Quantitative reverse transcription PCR
TEM: Transmission electron microscopy
VEGFA: Vascular endothelial growth factor-A
VPI: Very preterm infants
WB: Western blot

## References

[1] Blencowe H (2013). Born too soon: the global epidemiology of 15 million preterm births. Reprod. Health.

[2] Treyvaud, K. in *Seminars in Fetal and Neonatal Medicine.* 131–135 (Elsevier). 10.1016/j.siny.2013.10.008 24252709

[3] Maitre NL (2015). Respiratory consequences of prematurity: evolution of a diagnosis and development of a comprehensive approach. J. Perinatol..

[4] Stoll BJ (2015). Trends in care practices, morbidity, and mortality of extremely preterm neonates, 1993–2012. JAMA.

[5] Thébaud B (2019). Bronchopulmonary dysplasia. Nat. Rev. Dis. Primers.

[6] Abman SH (2001). Bronchopulmonary dysplasia: "a vascular hypothesis". Am. J. Respir. Crit. Care Med..

[7] Jobe AH (2015). Animal models, learning lessons to prevent and treat neonatal chronic lung disease. Front. Med. (Lausanne).

[8] Ferrara N, Davis-Smyth T (1997). The biology of vascular endothelial growth factor. Endocr. Rev..

[9] Klekamp JG, Jarzecka K, Perkett EA (1999). Exposure to hyperoxia decreases the expression of vascular endothelial growth factor and its receptors in adult rat lungs. Am. J. Pathol..

[10] He G-H (2021). Mesenchymal stem cell-derived exosomes inhibit the VEGF-A expression in human retinal vascular endothelial cells induced by high glucose. Int. J. Ophthalmol..

[11] Matthay, M. A. & Abman, S. H. Vol. 197 10–12 (American Thoracic Society, 2018).

[12] Kalluri, R. & LeBleu, V. S. The biology, function, and biomedical applications of exosomes. *Science* **367**, eaau6977 (2020). 10.1126/science.aau6977 PMC7717626 32029601

[13] Théry C (2018). a position statement of the International Society for Extracellular Vesicles and update of the MISEV2014 guidelines. J. Extracell. Vesicles.

[14] Braun RK (2018). Intraperitoneal injection of MSC-derived exosomes prevent experimental bronchopulmonary dysplasia. Biochem. Biophys. Res. Commun..

[15] Zhang, X. *et al.* Exosomes secreted by endothelial progenitor cells improve the bioactivity of pulmonary microvascular endothelial cells exposed to hyperoxia in vitro. *Ann. Transl. Med.* **7** (2019). 10.21037/atm.2019.05.10 PMC6614332 31355221

[16] Dinh P-UC (2020). Inhalation of lung spheroid cell secretome and exosomes promotes lung repair in pulmonary fibrosis. Nat. Commun..

[17] White AC, Lavine KJ, Ornitz DM (2007). FGF9 and SHH regulate mesenchymal Vegfa expression and development of the pulmonary capillary network. Development.

[18] White, A. C. *et al.* FGF9 and SHH signaling coordinate lung growth and development through regulation of distinct mesenchymal domains. (2006). 10.1242/dev.02313 16540513

[19] Frontini MJ (2011). Fibroblast growth factor 9 delivery during angiogenesis produces durable, vasoresponsive microvessels wrapped by smooth muscle cells. Nat. Biotechnol..

[20] Zhong XQ (2021). Umbilical cord blood-derived exosomes from very preterm infants with bronchopulmonary dysplasia impaired endothelial angiogenesis: Roles of exosomal MicroRNAs. Front. Cell. Dev. Biol..

[21] Bancroft, J. D. & Gamble, M. *Theory and practice of histological techniques*. (Elsevier Health Sciences, 2008).

[22] Schneider CA, Rasband WS, Eliceiri KW (2012). NIH Image to ImageJ: 25 years of image analysis. Nat. Methods.

[23] Matute-Bello G (2011). An official American Thoracic Society workshop report: features and measurements of experimental acute lung injury in animals. Am. J. Respir. Cell Mol. Biol..

[24] Shen J, Fu G, Jiang L, Xu J, Li L (2013). Effect of dexmedetomidine pretreatment on lung injury following intestinal ischemia-reperfusion. Exp. Ther. Med..

[25] Martin M (2011). Cutadapt removes adapter sequences from high-throughput sequencing reads. EMBnet. J..

[26] Kim D, Paggi JM, Park C, Bennett C, Salzberg SL (2019). Graph-based genome alignment and genotyping with HISAT2 and HISAT-genotype. Nat. Biotechnol..

[27] Liao Y, Smyth GK, Shi W (2014). featureCounts: an efficient general purpose program for assigning sequence reads to genomic features. Bioinformatics.

[28] Love MI, Huber W, Anders S (2014). Moderated estimation of fold change and dispersion for RNA-seq data with DESeq2. Genome Biol..

[29] Wu T (2021). clusterProfiler 4.0: A universal enrichment tool for interpreting omics data. The Innovation.

[30] Livak KJ, Schmittgen TD (2001). Analysis of relative gene expression data using real-time quantitative PCR and the 2− ΔΔCT method. Methods.

[31] Bishop-Bailey D (2011). PPARs and angiogenesis. Biochem. Soc. Trans..

[32] Zhao S, Luo G, Wu H, Zhang L (2019). Placental growth factor gene silencing mitigates the epithelial-to-mesenchymal transition via the p38 MAPK pathway in rats with hyperoxia-induced lung injury. Mol. Med. Rep..

[33] Willis GR (2018). Mesenchymal stromal cell exosomes ameliorate experimental bronchopulmonary dysplasia and restore lung function through macrophage immunomodulation. Am. J. Respir. Crit. Care Med..

[34] Teng L (2022). Exosomes derived from human umbilical cord mesenchymal stem cells accelerate diabetic wound healing via promoting M2 macrophage polarization, angiogenesis, and collagen deposition. Int. J. Mol. Sci..

[35] Zhang Y (2021). Exosomes derived from human umbilical cord blood mesenchymal stem cells stimulate regenerative wound healing via transforming growth factor-β receptor inhibition. Stem Cell Res. Ther..

[36] Xie K, Liu L, Chen J, Liu F (2019). Exosomes derived from human umbilical cord blood mesenchymal stem cells improve hepatic ischemia reperfusion injury via delivering miR-1246. Cell Cycle.

[37] Xie K, Liu L, Chen J, Liu F (2019). Exosomal miR-1246 derived from human umbilical cord blood mesenchymal stem cells attenuates hepatic ischemia reperfusion injury by modulating T helper 17/regulatory T balance. IUBMB Life.

[38] Xi Y, Ju R, Wang Y (2022). Mesenchymal stem cell-derived extracellular vesicles for the treatment of bronchopulmonary dysplasia. Front. Pediatr..

[39] Zhong, X. q. *et al.* Umbilical cord blood–derived exosomes from healthy term pregnancies protect against hyperoxia‐induced lung injury in mice. *Clin. Transl. Sci.* (2023). 10.1111/cts.13502 PMC10264931 36869608

[40] Willis GR, Kourembanas S, Mitsialis SA (2017). Toward exosome-based therapeutics: Isolation, heterogeneity, and fit-for-purpose potency. Front. Cardiovasc. Med..

[41] Nardiello C, Mižíková I, Morty RE (2017). Looking ahead: where to next for animal models of bronchopulmonary dysplasia?. Cell Tissue Res..

[42] Loering S, Cameron GJ, Starkey MR, Hansbro PM (2019). Lung development and emerging roles for type 2 immunity. J. Pathol..

[43] Stocks J, Hislop A, Sonnappa S (2013). Early lung development: lifelong effect on respiratory health and disease. Lancet Respir. Med..

[44] Sun LL, Li WD, Lei FR, Li XQ (2018). The regulatory role of microRNAs in angiogenesis-related diseases. J. Cell Mol. Med..

[45] Kong X (2020). Progesterone induces cell apoptosis via the CACNA2D3/Ca2+/p38 MAPK pathway in endometrial cancer. Oncol. Rep..

[46] Yin Y (2015). Fibroblast growth factor 9 regulation by microRNAs controls lung development and links DICER1 loss to the pathogenesis of pleuropulmonary blastoma. PLoS Genet..

[47] Dong N (2017). MicroRNA-182 prevents vascular smooth muscle cell dedifferentiation via FGF9/PDGFRβ signaling. Int. J. Mol. Med..

[48] Liu F (2018). MicroRNA-27a controls the intracellular survival of Mycobacterium tuberculosis by regulating calcium-associated autophagy. Nat. Commun..

