# Supplementary figures and images for: Umbilical cord blood exosomes from very preterm infants with bronchopulmonary dysplasia aggravate lung injury in mice
Source: Sci Rep. 2023 May 27;13:8648. doi: 10.1038/s41598-023-35620-8 (PMC10224930; doi:10.1038/s41598-023-35620-8)

## TSG101

BPD-exos Ctr

75Kd  
50Kd  
37Kd  
25Kd

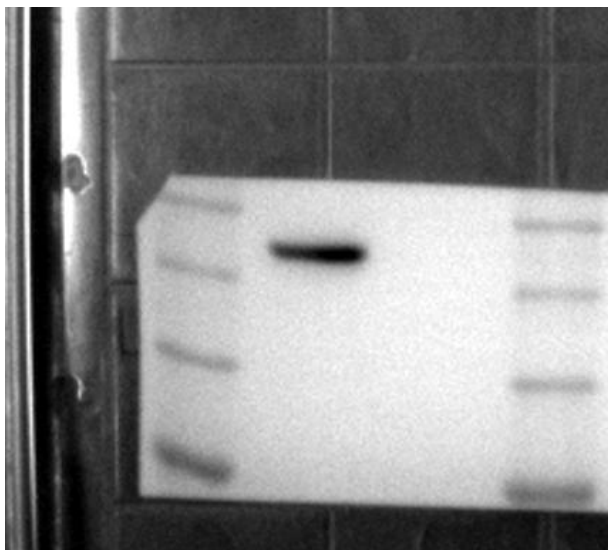

## Alix

BPD-exos Ctr

100Kd  
80Kd  
60Kd  
50Kd

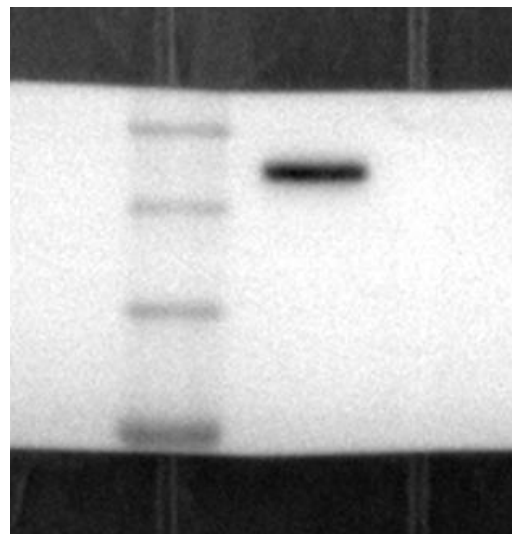

Supplement: Supplementary file 1 — Supplementary Information 1. [file 41598_2023_35620_MOESM1_ESM.pdf]
